# Psychological symptoms and brain activity alterations in women with PCOS and their relation to the reduced quality of life: a narrative review

**DOI:** 10.1007/s40618-024-02329-y

**Published:** 2024-03-15

**Authors:** J. Pinto, N. Cera, D. Pignatelli

**Affiliations:** 1 Faculty of Psychology and Education Sciences, University of Porto, 4200-135 Porto, Portugal; 2 Research Unit in Medical Imaging and Radiotherapy, Cross I&D Lisbon Research Center, Escola Superior de Saúde da Cruz Vermelha Portuguesa, Lisbon, Portugal; 3 Department of Endocrinology, Centro Hospitalar Universitário de São João, 4200-319 Porto, Portugal; 4 Faculty of Medicine, University of Porto, 4200-319 Porto, Portugal; 5 Department of Biomedicine, Faculty of Medicine at University of Porto, Porto, Portugal; 6 IPATIMUP Research Institute, Porto, Portugal

**Keywords:** PCOS, Quality of life, Brain alterations, Hyperandrogenism, EEG, fMRI

## Abstract

**Background:**

Polycystic ovary syndrome (PCOS) is the most common feminine endocrine disorder, characterized by androgen excess, ovulatory dysfunction, and polycystic ovarian morphology. The negative impact of symptoms on the quality of life (QoL) of patients is still not clear.

**Purpose:**

The present review aimed at studying the impact of the symptoms, the psychological symptoms, and brain alterations in women with PCOS.

**Methods:**

A systematic search was undertaken for studies that assessed the impact of PCOS symptoms on QoL, psychological symptoms, and brain alterations in PCOS patients.

**Results:**

Most of the information about QoL came from psychometric studies, which used culture-based questionnaires. Alterations of sleep quality, body image, and mood disorders can negatively affect the QoL of the patients. Sexual satisfaction and desire were affected by PCOS. Brain imaging studies showed functional alterations that are associated with impairments of visuospatial working memory, episodic and verbal memory, attention, and executive function.

**Conclusions:**

Several factors can negatively influence the quality of life of the patients, and they are directly related to hyperandrogenism and the risk of infertility. In particular, obesity, hirsutism, acne, and the fear of infertility can have a direct impact on self-esteem and sexual function. Metabolic and psychiatric comorbidities, such as mood, anxiety, and eating disorders, can affect the well-being of the patients. Moreover, specific cognitive alterations, such as impairments in attention and memory, can limit PCOS patients in a series of aspects of daily life.

## Introduction

Polycystic ovary syndrome (PCOS) is a multifaceted endocrine, reproductive, and metabolic condition [1, 2]. At present, PCOS is considered to be the most common feminine endocrine disorder during reproductive age [3] and is characterized by androgen excess, ovulatory dysfunction, and polycystic ovarian morphology (PCOM) [4]. The most recent international guidelines require the presence of two or more of the three previously mentioned criteria to establish a diagnosis of PCOS, after the exclusion of other related endocrine disorders [5]. The most common forms of presentation include irregular menstrual cycles and hirsutism [6], but acanthosis nigricans [7] and increased risk of infertility [8] should also be considered. According to the population of reference sample size and body mass index, the worldwide impact of PCOS varies from 5–15% [9] up to 20% of female patients of reproductive age [1].

PCOS is a complex syndrome associated with psychiatric comorbidities, neurocognitive and sexual alteration as shown by several studies performed during the last years. This condition can negatively affect the quality of life of the patients. Despite the wider use of the term quality of life (QoL) or health-related quality of life (HRQoL) in clinical research and medicine, there is no clear methodological and conceptual definition [10]. Recently, QoL was defined as a multidimensional outcome involving the physical, emotional, and socioeconomic well-being of one's life [11].

During the last few years, several studies have attempted to disentangle the negative impact of PCOS on several cognitive and emotional functions relevant to the quality of life of patients. Studies that used specific psychometric instruments assessed the impact and the presence of specific symptoms, providing information that can be useful, not only to assess the physical and emotional well-being in a specific culture but also to stimulate the implementation of new therapies and trials. Most of these instruments were adapted to different cultures.

Similarly, PCOS is comorbid with psychiatric disorders, such as anxiety and depression. The impact of these disorders on the quality of life of psychiatric patients, without other comorbidities, has been widely studied. Thus, studies providing information about neuropsychiatric symptoms in PCOS can allow us to characterize the dimensions of life that are more affected by the syndrome.

The present review aims to characterize the state of the art of the principal psychometric instruments that are useful to provide clinical information about the dimensions of life that can be affected in PCOS patients. Also, following what was mentioned above, studies about psychological symptoms, alterations in sexual behavior, and functional and structural brain alterations, as assessed by neuroimaging techniques, will be discussed.

## Materials and methods

Despite the narrative nature of the present review, our research question was based on the PICO strategy. Specifically, we were interested in analyzing the published literature that included studies involving PCOS women (P; Population), to study the symptoms and alterations as mentioned above (O; Outcome) with the use of questionnaires and imaging or psychophysiological techniques (I; Intervention) in comparison with healthy women (C; Comparison). To this purpose, we used terms related to the PICO strategy to perform an electronic search in the principal databases, such as MEDLINE, Scopus, and Web of Science.

To the same objective, previously published meta-analyses and narrative or systematic reviews were also retrieved to check the reported bibliographic references. In this way, the studies of interest have been retrieved and the content has been assessed.

### Diagnostic criteria

Due to the heterogeneous and multifactorial nature of PCOS symptoms, a lack of a clear, agreed-upon definition and diagnostic criteria is still present [12]. The diagnostic criteria have been recurrently changing, thus generating uncertainty on the type of PCOS that was considered in each study. The National Institutes of Health (NIH) proposed diagnostic criteria for PCOS, which included an association of chronic anovulation with androgen excess [13]. In 2004, in a meeting of the European Society of Human Reproduction and Embryology (ESHRE) with the American Society for Reproductive Medicine (ASRM), a new definition of PCOS was proposed. This definition is nowadays the most widely accepted. According to this consensus, two of the following criteria, the presence of hyperandrogenism, chronic ovulatory dysfunction, and ultrasound characteristics of polycystic ovaries, were relevant for diagnosis [14].

In 2006, the Androgen Excess Society (AES) proposed a different definition of PCOS as a disorder mostly characterized by androgen excess, e.g., the presence of clinical and/or biochemical hyperandrogenism together with ovulatory dysfunction, identified by either oligoanovulation or PCOM [8].

To maximize the comparability in research, the NIH Evidence-based Methodology Workshop Panel on PCOS recommended maintaining the comprehensive and inclusive diagnostic criteria of ESHRE/ASRM 2003 and classifying PCOS into four phenotypes [15]. Phenotypes A and B are often called “classic PCOS”, that is women with hyperandrogenism, ovulatory dysfunction, and with, in the case of phenotype A, or without, for phenotype B, PCOM. Phenotype C, or “ovulatory PCOS”, is distinguished by hyperandrogenism and PCOM without ovulatory dysfunction. In phenotype D, “non-hyperandrogenic PCOS”, women have ovulatory dysfunction and PCOM without hyperandrogenism.

### Psychological impact of PCOS

Since 2014, great attention has been paid to the comorbidities shown in PCOS [16]. PCOS women show increased odds of depressive and anxiety symptoms [16–20] (Table 1), in addition to a decreased QoL [18, 19, 21, 22] (Table 2). According to a recent meta-analysis, most of the published studies related to PCOS were focused on anxiety and depression [23]. Chaudhari et al**.** found that 38.6% of anxiety in their sample of PCOS is associated with infertility and alopecia, as well as 25.7% of depression being related to acne [24]. The assessment of depressive and anxious symptoms is very relevant. Nevertheless, the use of anxiety and depression-dedicated measures, such as Beck anxiety (BAI) [25] and depression inventories (BDI) [26], Hospital Anxiety and Depression Scale (HADS) [27], Hamilton Anxiety Rating Scale (Ham-A) [28] and Hamilton Depression Rating Scale (Ham-D) [29], seems to perform even better and may be preferable in the assessment of depression and anxiety in these patients.

**Table 1:** Principal findings about depression and anxiety in PCOS

Source	Design	Depression assessment tools	Anxiety assessment tools	Results
Blay et al. 2016	Systematic Review and Meta- Analysis	DSM-IV; HADS; BDI; PRIME-MD PHQ; TEMPS-A	DSM-IV; HADS; TEMPS-A	Six studies were included in the meta-analysis; of these, five reported the rates of anxiety and six provided data on the rates of depression. The rate of subjects with depressive and anxiety symptoms was higher in patients with PCOS compared to women without PCOS
Cooney et al. 2017	Systematic Review and Meta- Analysis	BDI; HADS; Zung self-rating depression scale; PHQ-9; Evaluation by Psychiatrist; SCL-90; BSI; BDI-SF; PRIME-MD PHQ; MADRS-S; MINI NPI by Psychiatrist: Any Major Depressive Episodes; CES-D; Screened with MMPI and Confirmed by psychologist – DSM IV; DACL State and DACL Trait Depression	BAI; HADS; SCL-90; BSI; PRIME-MD PHQ; BSA-S; MINI NPI by Psychiatrist: Generalized Anxiety; STAI; Screened with MMPI and Confirmed by psychologist – DSM IV ‘anxiety disorder’	Women with PCOS had increased odds of any depressive and anxiety symptoms, including moderate and severe symptoms in both conditions. In the meta-regression evaluating pooled SMDs between groups, women with PCOS and concurrent depression had higher mean values of age, BMI, hirsutism score and insulin resistance, while women with PCOS and concurrent anxiety had higher mean values of BMI, hirsutism score and free testosterone
Dokras et al. 2018	Systematic reviews and preparation of position statement	HADS; CES-D; MADRIS-S; Positive screen on Prime; HRDS; BDI	BSA-S; HADS; Positive screen on Prime; STAI	Several studies demonstrate that women with PCOS have an increased prevalence of higher depression and anxiety scores and higher odds of moderate and severe depressive and anxiety symptoms compared with controls. Obesity, hyperandrogenism, and fertility have a weak association with these symptoms. The few studies that have evaluated the impact of PCOS-related treatments (lifestyle interventions and pharmacotherapy) show no detrimental effect or some improvement in depressive and anxiety symptoms and HRQoL scores
Yin et al. 2021	Systematic Review and Meta- Analysis	BDI; HADS; SCL-90; SDS; BDI-S	HADS; SCL-90; SAS; STAI	The results of this study have indicated that women with PCOS suffer from depression, anxiety, and experience a lower quality of life, whereas their sexual function is not distinct from that of healthy women
Showkath et al. 2022	Case-controlled observational study	HADS	HADS	PCOS women showed significantly higher anxiety and depression scores along with low MoCA scores
Sheikh et al. 2023	Cross- sectional study	BDI	STAI	Non-white ethnicity PCOS reported higher rates of depression and lower BDD than white PCOS. PCOS born in India had higher anxiety and depression, but lower BDD rates than women born in the UK

*DSM-IV* Diagnostic and Statistical Manual of Mental Disorders, 4th Edition, *HADS* Hospital Anxiety and Depression Scale, *BDI* Beck Depression Inventory, *PRIME-MD PHQ* Primary Care Evaluation of Mental Disorders Patient Health Questionnaire, Temperament Evaluation of the Memphis, Pisa, Paris, and San Diego Auto questionnaire, *PCOS* polycystic ovary syndrome, *PHQ-9* Patient Health Questionnaire, *SCL-90* Symptom Checklist 90, *BSI* Brief Symptom Inventory, *MADRS-S* Montgomery Asberg Depression Rating Scale, *CES-D* Center for Epidemiological Studies-Depression, *MMPI* Minnesota Multiphasic Personality Inventory, *DACL* Depression Adjective Checklist, *BAI* Beck Anxiety Inventory, *BSA-S* Brief Scale for Anxiety, *STAI* State-Trait Anxiety Inventory, *SMDs* Standardized mean differences, *BMI* Body Mass Index, *HDRS* Hamilton Depression Rating Scale, *HRQoL* Health related quality of life, *SDS* Self-Rating Depression Scale, *SAS* Self-Rating Anxiety Scale, *MoCA* Montreal Cognitive Assessment, *BDD* Body dysmorphic disorder

**Table 2:** Quality of life in PCOS according to previous studies results

Source	Type of study	Results
Månsson et al. 2008	Multicenter study	Women with PCOS had higher lifetime incidence of depressive episodes, social phobia, and eating disorders than controls. Suicide attempts were seven times more common in the PCOS group than in the controls. Current as well as lifetime use of antidepressants and anxiolytic drugs were more common in the PCOS group
Dokras et al. 2018	Systematic review and preparation of position statement	HRQoL scores are consistently reduced in PCOS, with infertility and weight concerns having the most significant impact. Some studies suggest an increased prevalence of disordered eating in women with PCOS compared with controls. The few studies that have evaluated the impact of PCOS-related treatments (lifestyle interventions and pharmacotherapy) show no detrimental effect or some improvement in depressive and anxiety symptoms and HRQoL scores
Yin et al. 2021	Systematic review and meta-analysis	The results of this study have indicated that women with PCOS suffer from depression, anxiety, and experience a lower quality of life, whereas their sexual function is not distinct from that of healthy women
Bazarganipour et al. 2015	Systematic review and meta-analysis	The meta-analysis showed that the most affected domains in specific HRQOL were hirsutism and menstruation

*PCOS* polycystic ovary syndrome, *HRQoL* health related quality of life

However, Brucotao et al., highlighted the relevance of other psychiatric comorbidities, showing increased rates of obsessive–compulsive disorder and somatization in women with PCOS [23]. Apart from somatization disorders, a few studies reported the presence of bipolar and obsessive–compulsive disorders [30]. Interestingly, somatization disorders in PCOS can be related to physical symptoms and alteration of body appearance, such as in patients with hirsutism, which can create psychological distress. Some women with PCOS even reported feeling ‘different’ from other women and ‘less’ feminine’ [31]. PCOS also showed a higher lifetime incidence of social phobia and eating disorders [18, 21, 32] like bulimia nervosa and binge eating [33, 34]. Furthermore, PCOS reports higher rates of body dysmorphic disorder. Additionally, patients report poor body image, weight stigma, seven times more common chance of suicide, along with a higher current and lifetime use of antidepressants and anxiolytic drugs [21].

Moreover, a higher prevalence of sleeping disorders has been reported in PCOS [35], with disorders like hypersomnia and obstructive sleep apnea [34, 36, 37]. PCOS was associated with lower sleep quality in the presence of greater luteinizing hormone (LH) and serum insulin levels [38]. Indeed, higher LH levels were found to be significantly correlated with indices of poor sleep quality [39] and LH pulses occurred most frequently in association with brief awakenings, during periods of sleep [40]. Furthermore, in women with high BMI levels, sleep quality was also associated with insulin resistance, with a significantly lower total score of sleep quality in the insulin-resistant group than in the non-insulin-resistant group [41]. In both women with and without PCOS, insulin resistance was considered the strongest risk factor for sleep apnea, after controlling for age, BMI, and testosterone levels [42]. Similarly, insulin resistance markers are related to short sleep duration and even prior diagnosis of obstructive sleep apnea [43].

Psychiatric disorders represent one of the most common comorbidities in PCOS. Among these, anxiety and depression represent the most frequent, followed by eating and somatization disorders. However, recent studies also found an association between PCOS and sleep disorders.

### Clinical assessment and quality of life

The assessment of symptoms that can negatively affect the QoL is considered extremely relevant for the diagnosis and treatment of PCOS. Moreover, a few studies found that both NIH and non-NIH phenotypes of PCOS have exhibited similar psychological profiles. Milder phenotypes of the syndrome can still be associated with alteration in several cognitive domains [44]. Hence, psychological function and QoL should be taken into consideration in all PCOS patients [16].

Health-related quality of life (HRQoL) has been studied by some generic questionnaires, with the Short Form-36 questionnaire (SF-36) [45] being the most acknowledged and most often used instrument to measure HRQoL in various medical, including PCOS [46]. The results using S-36 have been fairly consistent in suggesting a reduction in HRQoL in PCOS patients. According to Bazarganipour et al., PCOS affected all domains in the SF-36, and psychological domains, such as the emotional and vitality ones, were the most negatively impacted by PCOS [47].

Other previous studies showed that the most affected domains in SF-36 were the ones related to emotional problems [48, 49].

Several specific psychometric questionnaires have been developed and validated to assess the QoL in PCOS women.

Cronin et al. developed a health-related quality-of-life questionnaire (PCOSQ) for women with polycystic ovary syndrome [50]. PCOSQ includes a total of 26 items and five subscales including emotions, body hair, weight, infertility, and menstrual problems. Later, the questionnaire was modified (M-PCOSQ) to include an acne subscale [50, 51].

In 2016, PCOSQ-50, developed by Nasiri-Amiri et al., is a new and comprehensive way of measuring QoL in PCOS women, including the sexual domain, which had been overlooked by previous questionnaires [52]. The PCOSQ-50 incorporated 50 items in a 5-point Likert scale representing six areas, namely emotion, obesity and menstrual disorders, fertility, sexual function, hirsutism, and coping.

According to Williams et al., a more sensitive PCOS quality-of-life measure was needed for clinical and research settings [53]. Therefore, they developed the 35-item polycystic ovary syndrome quality of life scale (PCOSQOL) that comprises the impact of PCOS, infertility, hirsutism, and mood subscales.

Nonetheless, even with the previous questionnaires, there was still an absence of a reliable and validated HRQoL questionnaire to measure the impact of PCOS on several life aspects of other cultures, religions, or ethnicities. This was the case for instance of Arabic women where, in particular, one would need to address sexuality in married women only. As such, Odhaib et al. developed two distinct questionnaires in Arabic for married and unmarried women with PCOS for effective QoL evaluation, PCOSQoL-47 and PCOSQoL-42 [54]. For married women with PCOS, PCOSQoL-47 comprehends five subscales, such as psychological and emotional status, fertility and sexual life, body image, hair disorders, and acne domain, as well as obesity and menstrual disorders. On the other hand, for unmarried women, PCOSQoL-42 also consists of five subscales, including the psychological and emotional domain, menstrual disorders and fertility domain, body image domain, hair disorders and acne domain, and coping domain**.** The M-PCOSQ was also adapted and validated for Iranian PCOS patients, which showed a prevalence of 11% in the total population [55]. Despite the Iranian validation confirming that the conceptualization of QoL is universal, the questionnaire was modified concerning the original version, with an item that was found loading in the menstruation disturbances scale instead of in the emotion subscale. These questionnaires may facilitate further studies in Arabic and Persian-speaking communities and other communities with similar social norms concerning marriage and sexuality.

Of all the HRQoL-specific questionnaires for PCOS, PCOSQ has been the most frequently used in most studies and clinical practice [44]. Both the PCOSQ and its modified variant showed adequate content and construct validity, reliability, and internal consistency [56]. Similar psychometric properties were shown in the German-adapted version, the PCOSQ-G [57]. In terms of structural validity, a few studies suggest that they have an additional dimension associated with menstruation, in addition to its existing dimensions [51, 58]. However, in a sample of Serbian women affected by PCOS, who participated in a validation study for PCOSQ-50 [59], the most relevant affected dimensions were hirsutism, obesity, and menstrual disorders. As stated by the authors, PCOSQ-50 is a useful and valid instrument to assess the HRQoL in women with PCOS, showing good psychometric properties.

A meta-analysis, which assessed the homogeneity of the scores of each domain of PCOSQ/MPCOSQ, found that the most significantly affected domains of HRQoL in PCOS were hirsutism and menstruation [60]. Whereas in the general population, hirsutism affects 4–11% of women, in PCOS, its prevalence is estimated to be 65–75% [9, 61].

Cultural factors are important variables that can be taken into consideration for the assessment of QoL. However, HRQoL may vary across populations according to the discrepancy in psychosocial factors such as cultural heritage, family structure, medical systems, and norms related to illness-related communication. Predictably, cultural variables seem to influence responses to different dimensions of symptoms. For instance, compared to Austrian women, Brazilian women demonstrated considerably more distress related to hirsutism, menstrual irregularities, and infertility [62], while Islamic immigrant women expressed significantly more concern about menstrual irregularities and infertility [63].

These results were confirmed by a cross-sectional study of 300 women with PCOS that found menstruation and infertility to be the HRQoL aspects more affected by PCOS [64]. Thus, in cultures in which there is a higher expectation of women having children, it would be expected that infertility would have a notably negative impact on QoL.

The assessment of quality of life and health-related quality of life in PCOS showed that specific and patient-based psychometric instruments developed in the last years are valid and reliable instruments. They can be useful in both clinical and research settings. Nonetheless, cultural differences need to be taken into account during the clinical assessment performed with one or more of the above-described scales.

### Sexuality and sexual behavior

The research on the psychosocial aspects of PCOS has only recently emerged, and data on sexual function in women with PCOS is exiguous and often contradicting. Female sexual function (FSF) is an intricate biopsychosocial phenomenon that can be influenced by many factors. It can be impaired by metabolic syndrome [65], obesity [66, 67], infertility [68], mental health [69–72], self-esteem [73, 74], body image [75], and body awareness [76].

These factors also occur frequently in PCOS and may also promote sexual dysfunction. Sexual dysfunction refers to difficulties that arise during the sexual response cycle which prevent the individual from experiencing satisfaction from sexual activity. Female sexual dysfunction (FSD) is a significant public health problem that affects 41% of premenopausal women [77]. Not only can high rates of sexual dysfunction be found in PCOS [78], but PCOS younger than 30 years are 40% more likely to have FSD than women without PCOS [79]. This frequent coexistence should not be neglected in clinical practice. In addition, a tendency was found for PCOS to report more dyspareunia and lack of sexual satisfaction [79].

As it is well known, androgens seem to play a considerable role in sexual desire (SD). Despite the role of modulation of sexual desire played by ovarian steroids, disagreement remains about the strength of the effect attributable to ovarian estradiol or testosterone in increasing SD in women [80].

Low levels of androgen showed a negative impact on SD [81–84]. However, it is still not clear if ovarian or adrenal androgens can play a different role in SD. In fact, besides having increased LH, as well as LH/FSH ratio, PCOS is characterized by high levels of dehydroepiandrosterone and androstenedione of adrenal origin as well as androstenedione and testosterone of ovarian origin [85].

PCOS and Nonclassic Congenital Adrenal Hyperplasia (NC-CAH) patients are both characterized by markedly increased production of androgens [86]. However, in NC-CAH, low sexual desire has been correlated with hirsutism and testosterone levels hence demonstrating that other factors besides androgens must also be active players in the development of sexual desire.

Although it is not easy to define blood androgen levels that can be considered as androgen deficiency, short-term clinical trials suggest that low-dose testosterone therapy in women with hypoactive sexual desire disorder can improve sexual function and satisfaction [82, 83]. Reciprocally, women suffering from hyperandrogenism can also benefit from the use of anti-androgenic oral contraception to improve sexual and social self-esteem [87].

Wåhlin-Jacobsen et al. found that in women between 25 and 44 years without systematic usage of hormonal contraception, SD was correlated with total testosterone, free testosterone, androstenedione, and dehydroepiandrosterone sulfate (DHEAS) [88]. In women aged between 45 and 65 years, SD was only associated with androstenedione. Nonetheless, clinical signs suggest that sensitivity to androgen levels, more than actual androgen levels, may be associated with some aspects of sexual desire [89].

According to Pastoor et al., PCOS women report substantially lower scores in sexual function, especially in arousal, lubrication, and orgasm scores [90]. PCOS women also indicated a greater decrease in terms of sexual attractiveness, satisfaction with their sex life, and a significant impact of their physical appearance and body hair on sexuality. Even though the arousal and lubrication subscales reported considerably inferior scores in the Female Sexual Function Index (FSFI), Zhao et al. found no substantial association between FSD and PCOS [91].

Therefore, it remains difficult to determine the association between androgens and sexual function in PCOS. Furthermore, menstrual irregularities and subfertility could also lead to loss of self-esteem and emotional distress, such as depression and anxiety, which could adversely affect the satisfaction of sexual relationships [92, 93]. Due to obesity and excess androgens, PCOS can find themselves less appealing because of dissatisfaction with their body and the potential loss of feminine identity [94]. Moreover, treatment with metformin for 6 months has reduced dyspareunia and improved sexual satisfaction and frequency attributable to increased insulin sensitivity [95]. This could be related to the known strong association of diabetes mellitus with FSD [96]. However, it can also be related to the positive impact that metformin use has in PCOS in terms of reducing body weight and BMI, total testosterone, androstenedione, fasting blood glucose, and increasing the likelihood of pregnancy [97], which leads to an increased QoL. Another hormone that plays a complex role in human sexuality is oxytocin [98].

Similarly, an interesting study that was done in a PCOS animal model demonstrated that endogenous/exogenous oxytocin showed positive results in the reduction of body weight. Indeed, this neuropeptide, synthesized in the hypothalamus, is involved in controlling metabolism, appetite, and body weight and the PCOS rats showed obesity and low levels of endogenous Oxytocin. After OT administration, PCOS rats showed a decrease in body weight [99]. These results can be promising, stimulate clinical trials in PCOS patients, and maybe result in an improvement of body image and sexual functions. Nevertheless, these results should be treated with caution since we have no information about the embodied image of rats. Despite this pioneering study, the polymorphisms of the gene of the Oxytocin receptor (OXTR) are associated with the development of insulin resistance and body weight [100, 101]. However, a recent population study reported five novel OXTR variants significantly associated with the risk of developing PCOS in multigenerational Italian families [102].

Women with excess androgens treated with anti-androgenic contraceptives experienced a significant improvement in hirsutism, sexual pain, orgasm, and satisfaction [87, 103]. Yet, other studies found no changes related to sexual desire levels even after normalizing androgen levels in PCOS [87].

Perturbations of sexual functions in PCOS involve the sexual satisfaction and desire dimensions. However, physical alterations, such as hirsutism or overweight and metabolic disorders can play a crucial role in sexual dysfunctions. Although these symptoms can be caused by hyperandrogenism, the role of androgens and anti-androgenic therapies in female sexual functions should be carefully assessed in future studies.

### Brain alterations in PCOS

Thanks to the recent advances in brain imaging techniques, such as structural (MRI) and functional magnetic resonance imaging (fMRI) or positron emission tomography (PET), it was possible to investigate the effect of hyperandrogenism on the female brain and related cognitive and emotional functions [104]. Several studies investigated gender differences in terms of cognition and emotion and simultaneously assessed brain responses. Similarly, during the last few years, electroencephalography (EEG) has provided complementary information about brain regions and the functions affected by hormonal alteration in PCOS. Although only a few studies investigated the brain alterations related to PCOS using neuroimaging techniques and EEG, several evidence found that PCOS is associated with poorer cognitive functioning (Table 3).

**Table 3:** Principal results as reported in functional brain imaging studies in PCOS

Source	*N* (Age ± SD)	Endocrinologic assessment	Psychological assessment	Neuroimaging technique	Tasks	Results
Marsh et al. 2013	7 IR- PCOS (25 ± 6) 5 HC (26 ± 8)	Free T; Total T; Fasting insulin; HOMA2-IR	BDI; STAI; PANAS; POMS	fMRI; PET	Emotional task (negative vs. neutral)	Patients with IR-PCOS had greater prefrontal and ventral anterior cingulate activation during an emotion task. No between-group differences with metformin therapy. Alterations in mu-opioid neurotransmission underlie limbic system activity and mood disorders in IR-PCOS
Li et al. 2020	41 PCOS (25.29 ± 3.15) 41 HC (26.22 ± 2.59)	T; FSH; LH; FINS; HOMA2-IR	SAS; BDI-II; PSQI	Rs-fMRI	2-Back task (WM); Stroop color word test (executive function); ANT (attention network task)	Enhanced FC between the left posterior cingulate gyrus and left inferior frontal gyrus was negatively correlated with the plasma LH level. These results may provide useful information regarding the potential mechanisms of cognitive impairment and emotional changes in this population
Lai et al., 2020	21 PCOS (25.0 ± 5.0)	P; E2; T; LH; FSH; PRL	–	Rs-fMRI	–	PCOS can induce changes in activities of brain regions responsible for visuospatial working memory, face processing and episodic memory. The reduced functional connectivity within the right frontal lobe is related with the high LH level in PCOS
Van Vugt et al. 2013	7 IR-PCOS (29.6 ± 6.19) 9 IS-PCOS (26.4 ± 5.27)	2 h OGTT G: I; HOMA; Fasting Glucose; FINS; T	9-item questionnaire to assess hunger and wellness	fMRI	Viewing pictures of high- calorie, low- calorie and controls pictures	The association between insulin sensitivity and corticolimbic responses to food pictures may reflect abnormal brain responses to insulin feedback that contribute to the development and or perpetuation of obesity in PCOS
Van Vugt et al. 2014	8 IR-PCOS (29.3 ± 5.80) 11 IS-PCOS (26.5 ± 4.72)	2 h OGTT G: I; HOMA 2-IR; Fasting glucose; FINS; T	9-item questionnaire to assess hunger and wellness (i.e., physical and mental state indicated by alertness, warmth, anxiety, thirst, dizziness and contentment)	fMRI	Viewing pictures of high- calorie foods and low- calorie foods after ingesting either 75 g glucose or an equivalent volume of water	Activity in anterior and posterior cingulate cortex including frontal and parietal regions, during a glucose challenge, correlated negatively with insulin sensitivity. No postprandial hyperinsulinemia inhibits brain responsiveness to food cues in insulin resistance, leading to greater non-homeostatic eating
Alsaadi and Vugt 2015	11 IR-PCOS (27.9 ± 5.84) 8 IS-PCOS (27.3 ± 4.65)	HOMA 2-IR; Fasting glucose; FINS; T	–	fMRI	Viewing pictures of food, following water or dextrose consumption	BOLD responses to food pictures were reduced during a glucose challenge in corticolimbic regions in insulin-sensitive subjects. Furthermore, the degree of insulin resistance positively correlated with the corticolimbic BOLD response in the medial prefrontal orbitofrontal cortex, anterior cingulate and ventral tegmental area in response to high-calorie pictures. BOLD signal in the orbitofrontal cortex, midbrain, hippocampus, and amygdala following a glucose challenge correlated with HOMA2-IR in response to HC-LC pictures
Soleman et al. 2016	14 PCOS (29.3 ± 5.6) 20 HC (25.6 ± 6.2)	A; DHEAS; LH; FSH; PRL; P; E2; T; SHBG; TSH; FAI PCOS started an antiandrogenic treatment after the first session combined with a hormonal oral contraceptive	National Adult Reading Test (NART) – Dutch version Hospital Anxiety and Depression scale (HADS)	fMRI	N-Back task (0- Back; 1-Back; 2- Back; 3- Back) – WM	At baseline, PCOS showed more activation than the control group within the right superior parietal lobe and the inferior parietal lobe during the task (all memory conditions). Task performance (speed and accuracy) did not differ between the groups. After antiandrogenic treatment the difference in overall brain activity between the groups disappeared and accuracy in the high memory load condition of the working memory task increased in women with PCOS
Lansdown et al. 2019	20 PCOS (29.80 ± 4.78) 20 HC (29.65 ± 4.96)	T; A; HbA1c; Insulin and Glucose during oral glucose tolerance test; HOMA-IR	–	fMRI	IFC (isometric forearm contraction) task	Brain activation during IFC was significantly greater in the PCOS in the right orbitofrontal cortex. Adjustment for insulin sensitivity abolished these between-group differences. This confirms sympathoexcitation in women with PCOS and an increased regional brain activation in response to IFC. The right orbitofrontal cortex BOLD signal change in women with PCOS is associated with insulin sensitivity
Showkath et al. 2022	37 PCOS (25.07 ± 3.00) 30 HC (24.38 ± 2.80)	–	BDI; STAI	EEG	MoCA (Executive, Naming, Attention, Language, Abstraction, Delayed Recall, Orientation) Visual oddball paradigm task	PCOS women showed significantly higher anxiety and depression scores along with low MoCA scores. Significantly reduced absolute alpha, increased frontal beta, and markedly increased theta (relative) power with increased TAR in the PCOS group were seen. Also, a significant reduction in P300 amplitude with prolonged latency during the visual oddball paradigm task was evident in them

*PCOS* polycystic ovary syndrome, *SD* standard deviation, *IR-PCOS* insulin resistant polycystic ovary syndrome, *HC* health controls, *Free T* free testosterone, *T* testosterone, *fMRI* functional magnetic resonance imaging, *Rs-fMRI* resting-state functional magnetic resonance imaging, *HOMA2-IR* homeostatic model assessment index for insulin resistance, *BDI* Beck Depression Inventory, *STAI* State-Trait Anxiety Inventory, *PANAS* Positive and Negative Affect Schedule, *POMS* Profile of Mood States, *PET* positron emission tomography, *FSH* follicle-stimulating hormone, *LH* luteinizing hormone, *FINS* fasting insulin, *SAS* Self-Rating Anxiety Scale, *BDI-II* Beck Depression Inventory II, *PSQI* Pittsburgh Sleep Quality Index, *WM* working memory, *FC* functional connectivity, *P* progesterone, *E2* estradiol, *PRL* prolactin, *IS-PCOS* insulin sensitive polycystic ovary syndrome, *2 h OGTT G:I* 2 h glucose to insulin ratio during an oral glucose tolerance tests, *ALFF* amplitude of low-frequency fluctuation, *DHEAS* dehydroepiandrosterone, *SHBG* sex hormone-binding globulin, *TSH* thyroid stimulating hormone, *FAI* free androgen index, *HbA1c* glycated hemoglobin, *EEG* electroencephalography, *MoCA* Montreal Cognitive Assessment, *TAR* theta/alpha ratio

According to Lai et al., PCOS induces functional alteration in brain regions responsible for visual working memory, such as the right middle and superior frontal gyrus [105]. The alteration found in these two frontal regions is also associated with a specific deficit of the attentional component of working memory. Similarly, the functional connectivity between the middle and superior frontal gyrus showed a negative correlation with LH levels and with the LH/FSH ratios. Notably, previous studies found that PCOS women had worse performance on visuospatial working memory [106]. In addition, more general visuospatial ability [107] and visuospatial learning [108] are negatively affected by PCOS. An fMRI study analyzed the effects of overexposure to androgens and subsequent antiandrogenic treatment on brain activity during working memory processing [109]. They reported a higher activation within the right superior and inferior parietal lobe in POCS during the task. After the treatment, no between-group difference in the global brain activity was found and PCOS showed better accuracy in high memory load conditions during working memory tasks. Thus, PCOS may need more neural resources during working memory tasks, having less efficient executive function [109].

These alterations in working memory confirmed previous findings showing episodic memory [110] and executive function [107] impairments in PCOS patients.

Li et al. also found higher executive functions and memory impairments [38]. Besides, since PCOS was associated with greater LH levels [38] (Table 4), they likewise found correlations between serum hormone alterations and cognitive function scores.

**Table 4:** Correlation between functional brain alterations and hormonal levels according to the fMRI and EEG studies reporting PCOS patients

Source	*N*	Age, years (SD)	BMI, kg/m^2^ (SD)	Free T pg/ml (SD)	T, ng/ml (SD)	LH, mIU/ml (SD)	FSH, mIU/ml (SD)	FINS, uU/ml (SD)	Correlations between functional properties and plasma hormone levels
Lai et al. 2020	PCOS (21)	25.0 (5.0)	24.1 (5.6)	–	0.63 (0.17)	12.36 (8.75)	5.86 (2.15)	–	Negative correlation between plasma LH level and FC in SFG.R and MFG.R (*r* = − 0.594, *p* = 0.005) LH/FSH ratio was negatively correlated with the FC between SFG.R and MFG.R (*r* = − 0.521, *p* = 0.015) No significant correlations were identified between the remaining hormone levels
	*NA*	–	–	–	–	–	–	–	
Van Vugt et al. 2013	IR-PCOS (7)	29.6 (6.19)	42.1* (9.23)	–	2.4 (0.90)	–	–	17.6 **** (1.85)	–
	IS-PCOS (9)	26.4 (5.27)	32.4 (7.94)	–	2.2 (0.91)	–	–	9.0 (3.87)	
Marsh et al. 2013	IR-PCOS (7)	25 (6)	35.3* (16.2)	1.5 ** (0.9)	0.70 (0.51)	–	–	14.4 ** (8.1)	-
	HC (5)	26 (8)	23.0 (3.1)	0.5 (0.2)	8.1 (0.9)	–	–	8.1 (0.9)	
Van Vugt et al. 2014	IR-PCOS (8)	29.3 (5.80)	41.2* (8.93)	–	2.5 (0.94)	–	–	18.8*** (3.61)	–
	IS-PCOS (11)	26.5 (4.72)	32.5 (7.75)	–	2.1 (0.91)	–	–	8.2 (3.87)	
Alsaadi and Van Vugt 2015	IR-PCOS (11)	27.9 (5.84)	40.0* (8.78)	–	2.2 (0.86)	–	–	17.8**** (3.61)	–
	IS-PCOS (8)	27.3 (4.65)	30.8 (7.03)	–	2.4 (0.91)	–	–	6.6 (1.99)	
Soleman et al. 2016	PCOS (14)	29.3 (5.6)	25.8 (5.6)	–	1.6 * (0.7)	11.0** (5.8)	5.4 (1.3)	–	No correlation between the levels of T and brain activity in the superior temporal lobe, the superior parietal lobe and the inferior parietal lobe during the first session
	HC (20)	25.6 (6.2)	24.5 (3.0)	–	1.1 (0.11)	4.0 (1.5)	5.4 (1.6)	–	
Lansdown et al. 2019	PCOS (20)	29.80 (4.78)	26.05 (4.90)	–	1.41 (0.77)	–	–	–	–
	HC (20)	29.65 (4.96)	26.11 (4.83)	–	1.03 (0.53)	–	–	–	
Li et al. 2020	PCOS (41)	25.29 (3.15)	24.62*** (4.88)	–	2.89*** (1.14)	11.90*** (7.78)	5.02 (1.47)	12.17*** (7.67)	The ALFF value in the MFG.L was negatively correlated with serum FINS (*r* = 0.536, *p* = 0.048) within subjects with IR. No significant correlation was found for subjects without IR Enhanced FC strength between MFG.L and IFG.L was positively correlated with T (*r* = 0.331, *p* = 0.034) Enhanced FC strength between PCG.L and IFGtriang.L was negatively correlated with LH (*r* = − 0.384, *p* = 0.012)
	HC (41)	26.22 (2.59)	20.31 (1.81)	–	1.71 (0.33)	4.28 (1.19)	5.05 (1.44)	6.25 (1.77)	
Showkath et al. 2022	PCOS (37)	25.07 (3.00)	27.39*** (3.85)	–	–	–	–	–	–
	HC (30)	24.38 (2.80)	21.87 (1.78)	–	–	–	–	–	

*PCOS* polycystic ovary syndrome, *SD* standard deviation, *BMI* body mass index, *Free T* free testosterone, *T* testosterone, *FSH* follicle-stimulating hormone, *LH* luteinizing hormone, *FINS* fasting insulin, *FC* functional connectivity, *SFG.R* right superior frontal gyrus, *MFG.R* right middle frontal gyrus, *IR-PCOS* insulin resistant polycystic ovary syndrome, *IS-PCOS* insulin sensitive polycystic ovary syndrome, *HC* health controls, *ALFF* amplitude of low-frequency fluctuation, *MFG.L* left middle frontal gyrus, *IR* insulin resistance, *IFG.L* left inferior frontal gyrus, *PCG.L* left posterior cingulate gyrus, *IFGtriang.L * Triangular part left inferior frontal gyrus

* =  < 0.05; ** = *p* < 0.01; *** = *p* < 0.001; **** = *p* < 0.0001

**Fig. 1:**
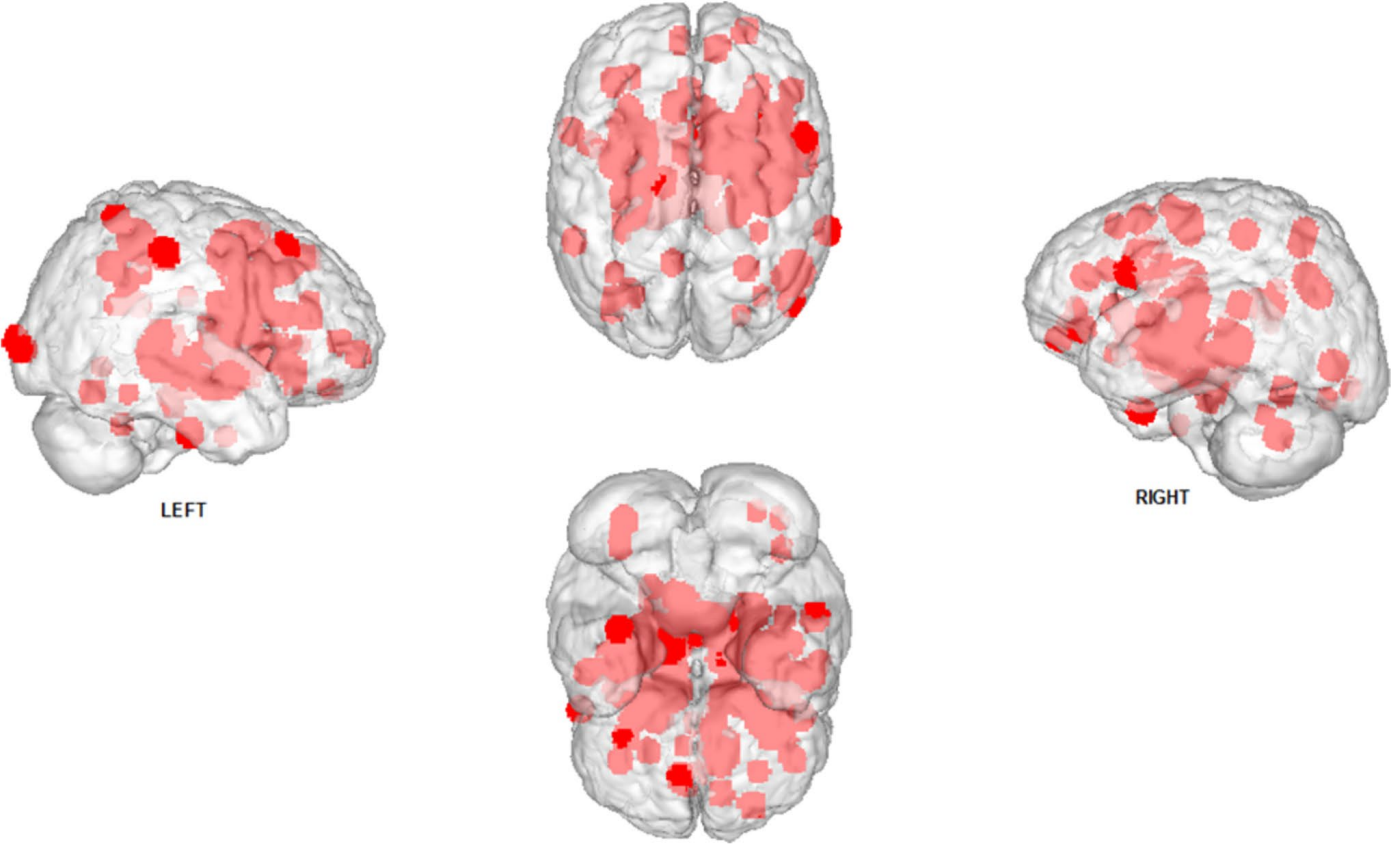
Resulting brain clusters from previously published fMRI studies on PCOS. The figure depicts a tridimensional map of the clusters as reported in previously published fMRI studies [38, 105, 109, 119–123]. The map was overimposed on an MNI (Montreal Neurological Institute) template in neurological convention. The map was created using Ginger Ale software (http://brainmap.org), and the 3D glass brain template was built using Mango software

A lower functional connectivity value, as measured using the amplitude of low-frequency fluctuation (ALFF), in the left middle frontal gyrus [38]. The left middle frontal gyrus is related to poor executive function and depressive disorders [38] and it showed a negative correlation with plasma insulin levels in insulin-resistant PCOS. A higher functional connectivity strength between the left middle and inferior frontal gyrus, which are correspondingly associated with the development of literacy [111] and language processing [112], was positively correlated with serum testosterone (free T) levels. It was also reported a negative correlation of LH plasma levels with a higher functional connectivity strength between the left posterior cingulate gyrus, a central node in the Default Mode Network [113, 114], and the pars triangularis of the left inferior frontal gyrus, which is related to language processing [115]. Finally, a higher functional connectivity strength was found with the right middle occipital gyrus associated with visuospatial processing [116], and the right inferior occipital gyrus, which is associated with impaired memory [38] (Fig. 1).

PCOS women have shown significantly lower performance on the tests of attention [107] and psychomotor speed than healthy women [108].

PCOS patients showed decreased neuropsychological performance scores [20] as measured by the Montreal Cognitive Assessment (MoCA) [117]. Then, participants showed less absolute alpha power, more frontal beta waves, during an EEG study, and significantly more theta relative power, with a higher theta-alpha ratio compared to controls. The fewer alpha waves and more theta activity with an increased theta-alpha ratio represent poor neural processing ability. These results indicate that PCOS even without any comorbidities can present subclinical cognitive impairment.

The performance of verbal fluency, verbal memory, and manual dexterity was shown to be negatively impacted by PCOS [118]. However, no differences were observed in mental rotation, spatial visualization, spatial perception, or perceptual speed. This should indicate that women with PCOS have poor performance on cognitive tasks that usually show a female advantage [118]. Even after a 3-month treatment of anti-androgen plus estradiol resulting in a hormonal reduction of free T levels, PCOS did not exhibit a substantial difference in performance on tests compared to pretreatment scores, apart from verbal fluency [106].

Despite several studies investigating the brain underpinnings of executive functions in PCOS, the cerebral correlates of emotional alterations are still partially undisclosed. Interestingly, the emotional alterations taken into consideration were related to metabolic issues shown by PCOS patients [119]. An association between insulin sensitivity and corticolimbic responses to food pictures was found by comparing high-calorie and low-calorie food pictures using fMRI [120]. The results suggested that this may reflect abnormal brain responses to insulin feedback and contribute to the development and/or perpetuation of obesity in PCOS. Afterward, Van Vugt et al. and Alsaadi and Van Vugt concluded that normal inhibition of corticolimbic brain responses to food pictures during a glucose challenge is compromised in insulin-resistant subjects [121, 122]. They also reported that higher brain responsiveness to food pictures during postprandial hyperinsulinemia might lead to higher non-homeostatic eating and perpetuate obesity in insulin-resistance subjects.

Lastly, according to Lansdown et al., not only was the brain activation in the right orbitofrontal cortex during isometric forearm contraction significantly higher in PCOS but this change was associated with insulin sensitivity [123].

Recent evidence from MRI studies reported structural brain alterations in PCOS. Decreased total brain volume and total gray matter (GM) were observed in PCOS patients [124]. These results were accentuated in obese PCOS when compared with lean PCOS women [125]. PCOS affected by obesity showed decreased GM volume in the caudate nucleus, ventral diencephalon, and hippocampus when compared to healthy controls. On the other hand, lean PCOS patients showed a decreased volume in correspondence of the amygdala. The hormonal changes in PCOS can induce hypertrophy and an increase in the volume of structures such as the pituitary gland. Following this hypothesis, Unlu and colleagues [126] found that the volume of the pituitary gland was significantly increased in a group of 26 PCOS without obesity, and the pituitary volume was positively correlated to the LH/FSH ratio. This result was partially confirmed in a subsequent study that did not observe a correlation between pituitary volume and hormonal values [127].

Other imaging studies showed specific alterations in the white matter (WM) in several brain districts [128, 129]. In a pioneer study, Udiavar and colleagues found decreased mean diffusivity at the level of the genu of the corpus callosum and right cingulum [110]. These alterations, according to the authors, were not related to BMI and pre-PCOS cognitive alterations. However, PCOS showed specific alteration in the white matter involving the insula, and thalamus and extending to the dorsolateral frontal and middle temporal cortex [130]. These WM alterations, positively associated with the levels of free T in PCOS, can suggest the increase in the extracellular space since the Diffusion Weighted images (DWI) were assessed by the apparent diffusion coefficient (ADC). ACD is considered a reliable index to assess the presence of pathological tissue abnormalities in the parenchyma, such as vasogenic edemas [131]. However, in a quantitative study assessing the WM microstructures, no differences were found between PCOS and healthy women [132]. Despite the absence of between-group differences, the authors found a correlation between fractional anisotropy (FA), axial diffusivity (AD), and serum testosterone. In PCOS, insulin resistance is associated with an increase in AD that can be considered an index sensitive to axonal fiber factors [133]. In a recent tractography study, PCOS showed a higher level of structural connectivity than healthy women in the left infundibular region that connects the arcuate nucleus and median eminence [134]. In the same study, the authors assessed the integrity of the hypothalamic membrane metabolism and the glial axonal signaling measuring metabolites, such as N-acetyl aspartate (NAA) and choline (Cho), normalized with the creatine (Cr), using Magnetic Resonance Spectroscopy (MRS). NAA/Cr was significantly higher in the PCOS women than in controls showing a disruption of hypothalamic neuroplasticity. No significant between-group differences were found for Cho/Cr. NAA is a metabolite and an important marker of neuronal functioning [135]. Its variations are usually related to osmoregulatory stress and brain disorders [136].

Functional brain imaging studies highlighted the impact of PCOS on a wider set of brain regions, mainly involving frontal, temporal, and subcortical regions. Functional changes were related to impairments in cognitive functions, such as working and episodic memory and attention. These cognitive impairments were also confirmed by neuropsychological studies that reported decreased performance during the assessment in women with PCOS. Appetitive behaviors and their brain underpinnings were also altered by PCOS. Few and most recent studies investigated the abnormalities in the white and gray matter. Most of them agreed with finding decreased gray matter volume in PCOS. However, hormonal changes observed in PCOS are related to an increased volume of the pituitary gland and neurochemical alteration in patients.

## Discussion

PCOS represents one of the most complex and multifaceted disorders and its consequences are evident in many aspects of the life of the patients. PCOS facets are reflected not only in the diagnostic criteria, but also in studies analyzing many aspects of the disease.

Despite the numerous studies that investigated the affected quality of life dimensions, processes and mechanisms underlying PCOS are still partially unknown. Indeed, according to the Bazarganipour model [137], the health-related quality of life in PCOS women is affected by clinical and psychological variables. However, the most prominent clinical variable is hirsutism and infertility which is related to psychological distress, self-esteem, sexual functions, and body image. These variables are in direct and reciprocal relationships, showing the complexity of the psychological alteration found in women with PCOS. As reported in the present review, this phenomenon invades other functions that can affect the quality of life. Although the alteration in body image and the distress that accompanies it, due, for example, to hirsutism and obesity or weight excess, are relatively well characterized, other recent studies showed specific cognitive impairment affecting executive functions and memory. Few studies have investigated cognitive impairment and its cerebral correlates in PCOS patients using in vivo brain imaging techniques. These pioneering studies focused their attention on the substrate of episodic memory and working memory. These specific alterations of memory are related to impaired attention and concentration and are common in cases of stress. Besides, they were previously observed in Post Traumatic Stress Disorder (PTSD) and veterans [138]. In this way, it is possible that memory impairment can be related to psychological distress experienced by PCOS. Nonetheless, psychiatric comorbidities can also affect cognitive functions and cerebral responses in these patients. Most of the patients experience depression, somatization, and bipolar disorder. Mood disorders can affect cognitive functions, such as sustained attention [139] and working memory [140]. Therefore, it is not easy to establish if a specific brain response or cognitive alteration is related to PCOS or is related to comorbidities or stress-related symptoms. However, cognitive impairments, such as impairment of attention or working and episodic memory, need to be taken into consideration for the assessment of the quality of life in these patients.

Similarly, most of the functional brain imaging studies investigated brain alterations underlying working memory and food-related stimuli presentation. No published studies assessed the brain correlates of sexual arousal or desire using fMRI or EEG in women with PCOS, comparing them with normal women or women affected by Hypoactive Sexual Desire Disorder.

Alterations of sexual behavior have been frequently identified in women with PCOS, with decreased levels of satisfaction and desire. The assessment of Quality of Life in these patients should also include the assessment of sexual satisfaction, desire, and orgasm. Therefore, the assessment of QoL should also take into account socio-cultural differences and the context in which the patients lived. For this reason, a mixed approach with an anamnestic interview with neuropsychological and psychological testing is advisable.

Moreover, a few recent studies [99] reported the role played by other hormones, such as GLP1, AMH, leptin, insulin, arginine vasopressin, and oxytocin in PCOS. Since their relevance in metabolic and cardiovascular risk [141] as well as in socio-cognitive functions, oxytocin and arginine vasopressin, in particular, need to be taken into account for future studies. In particular, increased plasma levels of arginine-vasopressin have been found in patients affected by depression [142] and depressive mood is very common in PCOS patients.

## Conclusion

Several factors can negatively impact the quality of life of women suffering from PCOS. Most of them are directly related to the clinical condition due to hyperandrogenism and the risk of infertility. Symptoms, such as obesity, hirsutism, acne, and the fear of infertility can have a direct impact on self-esteem and also in sexual function. Metabolic and psychiatric comorbidities can affect the well-being of these patients. Moreover, specific cognitive alterations, such as impairments in attention and working memory, can limit PCOS patients in a series of aspects of daily life.

## Data Availability

Not applicable.
